# The epidemiological characteristics and spatio-temporal analysis of childhood hand, foot and mouth disease in Korea, 2011-2017

**DOI:** 10.1371/journal.pone.0227803

**Published:** 2020-01-13

**Authors:** Soojin Baek, Seongwoo Park, Hye Kyung Park, Byung Chul Chun

**Affiliations:** 1 Department of Public Health, Korea University Graduate School, Seoul, Korea; 2 Division of Strategic Planning for Emerging Diseases, Korea Centers for Disease Control and Prevention, Chungcheongbuk-do, Korea; 3 Department of Preventive Medicine, College of Medicine, Korea University, Seoul, Korea; Institut Pasteur, FRANCE

## Abstract

**Objectives:**

Hand-foot-mouth disease (HFMD) is a common viral infection in children, with a significant disease burden due to its high contagion rate. This report studied the epidemiological characteristics as well as the chronological and geographical distribution of HFMD in children younger than 6 years of age in Korea.

**Methods:**

This report established a database by integrating population and geographical data from health insurance claims for HFMD between 2011 and 2017, with an age restriction of ≤6 years, and explored the epidemiological characteristics of both HFMD patients and hospitalized cases in Korea. The relative risk ratio and spatio-temporal scan statistics were calculated by administrative district, using SaTScan.

**Results:**

Over a 7-year period, 1,879,342 children under the age of 6 were diagnosed with HFMD (8.4 of 100 persons younger than 6 years of age). Seasonal incidence tended to increase from week 17 (May) peak between weeks 29 (July) and 39 (September), and increase rapidly in 1- to 2-year cycles. HFMD primarily occurred in children younger than 4 years of age. Furthermore, the greatest proportion of cases occurred at ages 1 (39.2%) and 2 (25.7%). Overall, 92.6% of all cases occurred before the age of 6. The proportion of cases before the age of 6was slightly higher in males. The timing of HFMD epidemics differed over the years. In 2015, the HFMD cumulative incidence was the lowest (5.5/1,000), and the spatio-temporal cluster (RR 2.32) was predominantly located south-central Korea, covering 65 counties for twenty-two weeks. In 2016, however, its cumulative incidence was high (RR 6.34) over a short period (11 weeks) in specific areas such as Ulsan, Daegu, Busan, and Gyeongnam. Also, the southern parts of Korea were found to have a higher rate of hospitalization.

**Conclusions:**

HFMD in Korea is common in children younger than 6 years of age, and it tends to peak in the summer.

## Introduction

Hand-foot-mouth disease (HFMD) is a common viral infection in children younger than 6 years of age. The main causative pathogens for HFMD are the enterovirus Coxsackievirus A16 and enterovirus 71 [1, 2]. Most cases show mild clinical symptoms; However, although rare, neurological complications can occur that can lead to death [3]. Infection occurs through direct contact or droplets, via respiratory secretions such as saliva, sputum, and nasal discharge, and from excretory materials from an infected person [4, 5]. Infection commonly occurs in an environment where many children gather in a given area (i.e. nursery school or playgrounds). HFMD has a dormant period of 3–7 days [4]. Given that there is more than one causative virus for HFMD, reinfection through another viral strain is possible even after recovery. Although an EV71 vaccine has been developed and recently introduced in China, the vaccine’s availability is limited to China, and only symptomatic treatment is available in other countries. There are no specific treatment options, such as antiviral or pharmacological therapy. The most effective preventative measure remains good personal hygiene, disinfection of toys and household goods, and thorough washing of clothes contaminated with an infected person’s excretory materials [3, 4].

In June 2009, the Korea National Institute of Health (NIH) designated HFMD as a national infectious disease that requires reporting by sentinel sites through a sentinel surveillance system. Surveillance data are directly reported from sentinel sites to the Korea Center for Disease Control (KCDC) Division of Infectious Disease Surveillance through the web reporting system on a weekly basis. [3]. In Korea, HFMD incidence peaks in the summer (June-July), In 2016, the highest incidence occurred in week 26 (June 19–25), with 51.1 suspected cases of HFMD in 1000 outpatients. and the 4 fatal cases of enterovirus 71, which can cause more severe neurological complications. To date, no study has been performed on the epidemiological characteristics or geographical incidence of HFMD, apart from those that have studied seasonal tendencies in Korea.

This report aimed to explore the epidemic pattern of HFMD in Korea by investigating the epidemiological characteristics of HFMD in children younger than 6 years of age, as well as its temporal and geographical distribution.

## Materials and methods

### Study area

The Republic of Korea (hereafter ‘Korea’), situated at latitude 33°N to 38°N and longitude 126° E to 131° E, is located in northeast Asia. It comprises an estimated 51.4 million residents distributed over 100,363 km^2^. Korea has a temperate climate with four distinct seasons with the coldest months lasting from December to March and the warmest months falling between June and August.

### Data

This report obtained data from the South Korea Health Insurance Review and Assessment Service (HIRA), which maintains health insurance population- based longitudinal databases that encompass approximately 97% of the people living in Korea (https://www.hira.or.kr/eng/). Medical expenses claimed between January 1, 2011 and December 31, 2017 under the main diagnostic code of HFMD (KCD-10 code ‘B08.4’) at health care institutions (excluding dentists, midwives, psychiatrists, and traditional medical practitioners) or public health centers for children younger than the age of 6 were used for the data analysis.

### Definition of an HFMD case

Due to the risk of reinfection with another viral strain in HFMD, the treatment-free period was defined as 30 days, with return visits for confirmation of eradication considered a single disease episode.

## Methods

For the population standardization of HFMD, the cumulative incidence per 100 persons in a given administrative district was calculated in 1-year age units for a given period, using resident population data

This report analyzed the spatiotemporal clustering of the incidence of HFMD in Korea between 2011 and 2017 at the municipal level by using Kulldorff’s spatial scan statistics via SaTScan 9.6 [6]. The relative risk (RR) ratio for each administrative district was calculated using the number of cases of HFMD during the study period and the total population size. The relative risk ratio is the value obtained by dividing the predictive value within a cluster by the predictive value outside a cluster.

$$RR=\frac{n/E[n]}{\left(N-n)/(E[N]-E[n]\right)}=\frac{n/E[n]}{\left(N-n)/(N-E[n]\right)}$$

Where n is the number of observed cases within the cluster and N is the total number of cases in the data set. Note that since the analysis is conditional on the total number of cases observed, E[N] = N. To identify clusters that are considered high HFMD risk, spatio-temporal scan statistics were used. SaTscan scans gradually across time and/or space to identify possible clusters by comparing the number of observed incidences and expected incidences (assuming random distribution) inside the window at each location. The null hypothesis is that the risk of HFMD is randomly distributed throughout the study area, while the alternative hypothesis is that the risk increases inside the window more than in areas outside of at least one circle or cylinder. The log-likelihood ratio (LLR) is the hypothesis-testing statistic estimated based on Monte Carlo randomization.

The log-likelihood ratio in Poisson distribution is computed as:

$$LLR={\log\left(\frac{n}{E[n]}\right)}^{n}{\left(\frac{N-n}{N-E[n]}\right)}^{N-n}I()$$

Where N is the total number of cases, n is the observed number of cases within the window and E[n] is the covariate adjusted expected number of cases within the window under the null-hypothesis. Note that since the analysis is conditional on the total number of cases observed, N-E[n] is the expected number of cases outside the window. I() is an indicator function. The window with the maximum likelihood ratio is the most likely cluster; In order words, it identifies the cluster that is least likely to occur by chance. In addition to the most likely cluster, SaTScan also designates secondary clusters for purely spatial and spatiotemporal analyses and ranks them according to their estimated LLR statistic. SaTScan scans for clusters by using different criteria; the criterion recommended by SaTScan is the percentage of the population at risk, with a value of 50% [6]. This report tested the percentage of the population at risk from 10% to 50%, and from the result, 20% performed best; In order words, the value of 20% did not include neighboring cities that have a non-elevated risk.

The largest size of a cluster is 20% of the total population, and the longest timeframe for a cluster is 6 months. An analysis was performed using 999 Monte Carlo simulations [7]. The geographic unit for analysis was 250 administrative districts, and the temporal unit was 366 weeks, between 2011 and 2017. The analysis was performed weekly, to adjust for the day-of-the-week effect of medical expense claims.

SaTScan 9.6 software was used to perform the spatiotemporal cluster analysis of HFMD and QGis 2.18 software was used to illustrate the results.

### Ethics statement

This study used public health big data provided by the Health Insurance Review and Assessment Service (HIRA) and was conducted with the approval of the Korea University Institutional Ethics Committee. Consent was waived as per Institutional Review Board approval (IRB no. KU-IRB-18-EX-127-A-1).

## Results

The number of cases diagnosed as HFMD in children younger than 6 years of age between 2011 and 2017 was 1,879,342 (8.4 of 100 persons younger than 6 years of age). The proportion of male (53.5%) among HFMD cases was slightly higher compared with females, and 3% of the total patient population (both male and female) were hospitalized. The proportion of cases was higher in children younger than 3 years of age, and decreased gradually from age 4. The greatest proportion of cases occurred at ages 1 [39.2% (32.7–44.1%)] and 2 [25.7% (23.9–28.2%)]. Overall, 92.6% of all cases occurred before the age of 6 (Table 1).

**Table 1:** The epidemiological characteristics of HFMD cases (%) in Korea, 2011–2017.

Year	Total	2011	2012	2013	2014	2015	2016	2017
Total	1,879,342(100)	309,507(100)	187,110(100)	246,306(100)	328,479(100)	175,507(100)	421,279(100)	211,154(100)
Inpatient	54,175(2.9)	5,949(1.9)	5,339(2.9)	7,615(3.1)	9,168(2.8)	5,462(3.1)	13,781(3.3)	6,861(3.2)
Outpatient	1,825,167(97.1)	303,558(98.1)	181,771(97.1)	238,691(96.9)	319,311(97.2)	170,045(96.9)	407,498(96.7)	204,293(96.8)
Gender								
Male	1,004,635(53.5)	164,361(53.1)	100,467(53.7)	132,369(53.7)	177,596(54.1)	93,343(53.2)	224,173(53.2)	112,326(53.2)
Female	874,707(46.5)	145,146(46.9)	86,643(46.3)	113,937(46.3)	150,883(45.9)	82,164(46.8)	197,106(46.8)	98,828(46.8)
Age (year)								
0–1	736,130(39.2)	101,309(32.7)	83,060(44.4)	101,237(41.1)	123,854(37.7)	73,842(42.1)	159,640(37.9)	93,188(44.1)
2	483,340(25.7)	76,541(24.7)	44,638(23.9)	62,307(25.3)	92,627(28.2)	47,168(26.9)	106,272(25.2)	53,787(25.5)
3	307,672(16.4)	60,693(19.6)	27,057(14.5)	36,695(14.9)	55,549(16.9)	27,539(15.7)	70,782(16.8)	29,357(13.9)
4	180,944(9.6)	38,057(12.3)	16,888(9.0)	22,259(9.0)	28,466(8.7)	14,440(8.2)	43,366(10.3)	17,468(8.3)
5	108,900(5.8)	20,907(6.8)	9,891(5.3)	15,165(6.2)	17,342(5.3)	7,820(4.5)	26,816(6.4)	10,959(5.2)
6	62,356(3.3)	12,000(3.9)	5,576(3.0)	8,643(3.5)	10,641(3.2)	4,698(2.7)	14,403(3.4)	6,395(3.0)

**Fig 1:**
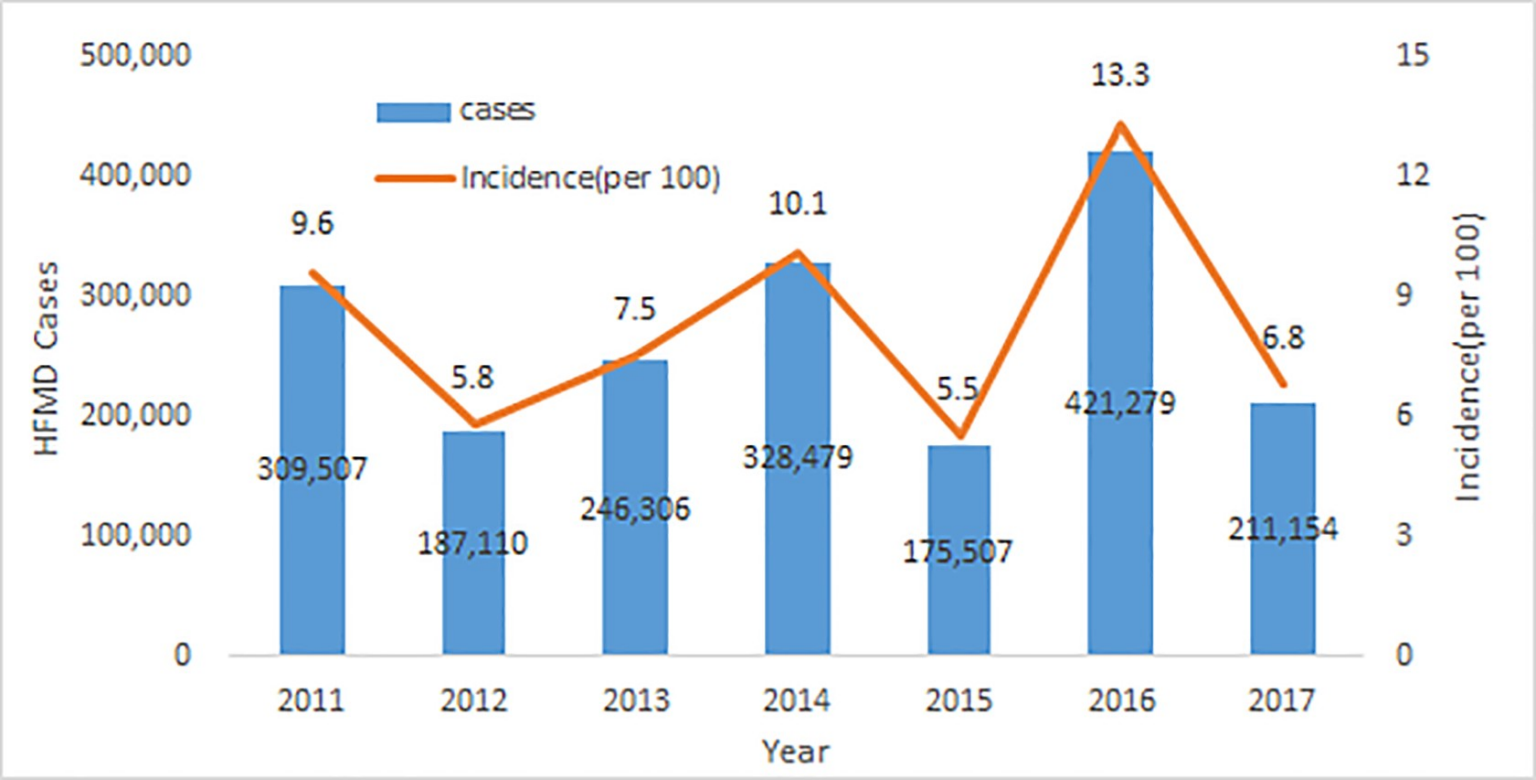
Annual trends of HFMD cases and cumulative incidence in Korea, 2011–2017.

The annual cumulative incidence showed an increasing and decreasing pattern. The cumulative incidence decreased in 2012 compared with 2011, and increased again in 2013–2014. The cumulative incidence was lowest in 2015 and peaked in 2016 before decreasing again in 2017. In 2016, when the HFMD cumulative incidence was highest, the incidence rate per 100 persons was 18.8 among those younger than 1 year of age and 24.2 among those between 1–2 years of age, totaling over 20% of children younger than 2 years of age. The ratio of hospitalized patients was also relatively high (Table 2, Fig 1).

**Table 2:** The epidemiological characteristics of HFMD cumulative incidences in Korea(per 100 persons), 2011–2017.

Year	Total	2011	2012	2013	2014	2015	2016	2017
**Total**	8.4	9.6	5.8	7.5	10.1	5.5	13.3	6.8
**Inpatient**	0.2	0.2	0.2	0.2	0.3	0.2	0.4	0.2
**Outpatient**	8.1	5.6	5.6	7.3	9.9	5.3	12.9	6.6
**Gender**								
**Male**	8.7	9.9	6.0	7.9	10.7	5.7	13.8	7.1
**Female**	8.0	9.3	5.5	7.2	9.6	5.3	12.8	6.6
**Age (year)**								
**0–1**	12.0	11.2	8.9	11.0	14.0	8.6	18.8	11.7
**2**	15.1	16.8	9.7	13.2	19.3	10.2	24.2	12.2
**3**	9.4	12.6	5.9	8.0	11.7	5.7	15.2	6.7
**4**	5.5	8.1	3.5	4.9	6.2	3.0	9.0	3.8
**5**	3.3	4.7	2.1	3.2	3.8	1.7	5.7	2.3
**6**	1.9	2.6	1.3	1.8	2.2	1.0	3.1	1.3

**Fig 2:**
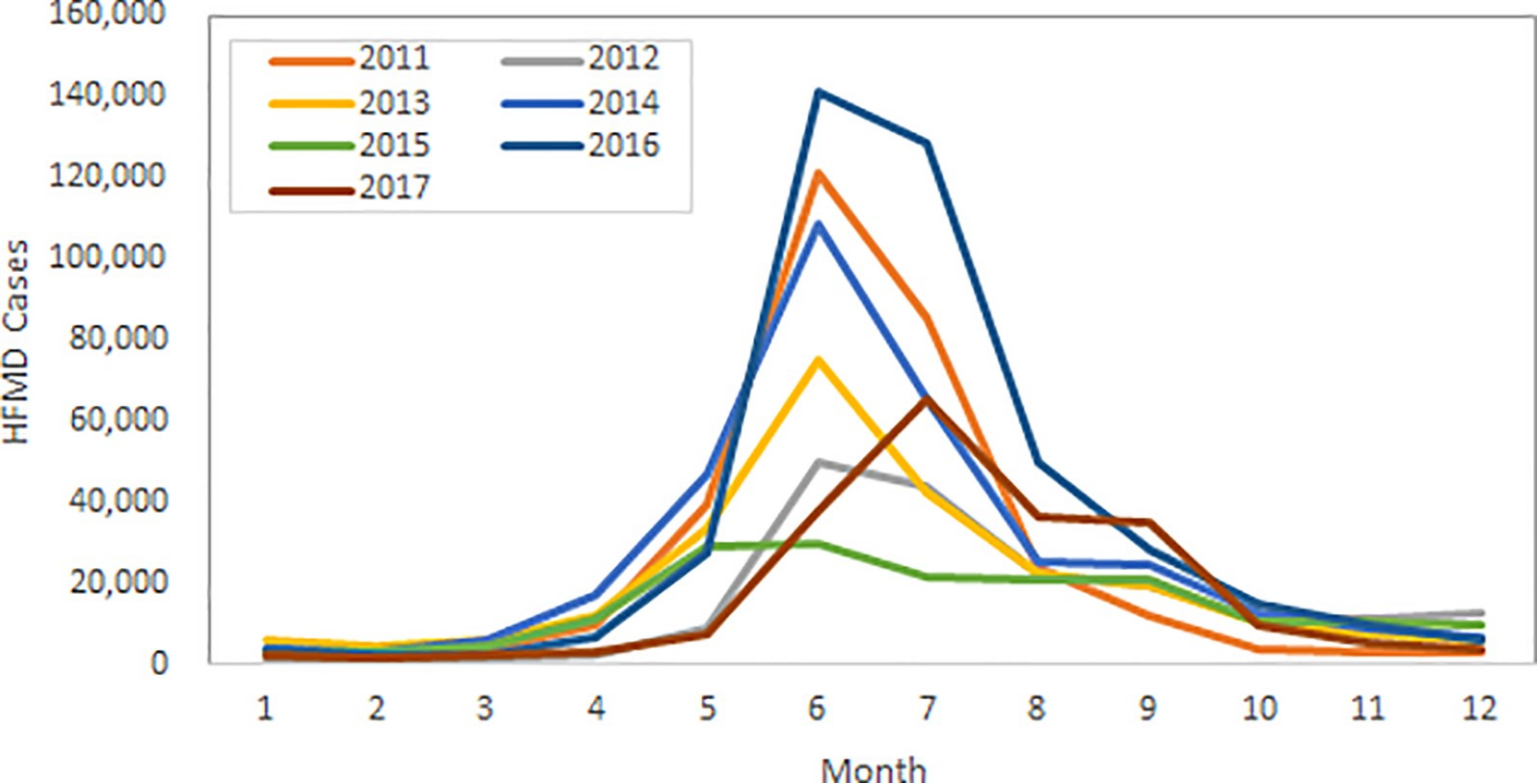
Monthly trends of HFMD cases in Korea, 2011–2017.

HFMD in Korea shows a seasonal pattern, with cases increasing in May and peaking in June (Fig 2).

**Fig 3:**
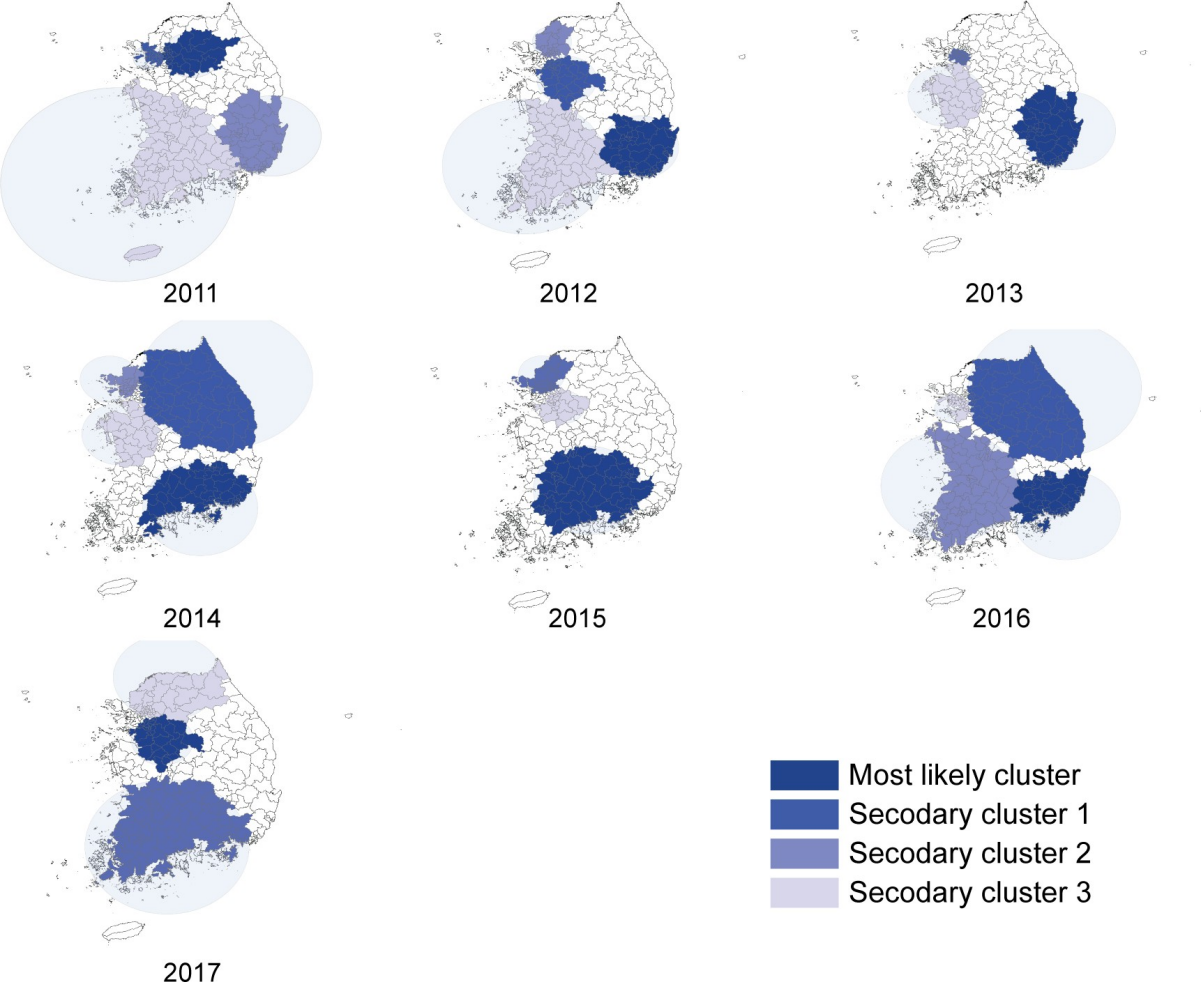
Spatiotemporal clusters of HFMD 2011–2017, setting 20% as the maximum cluster size.

Spatial and temporal analyses were performed to explore the timing and location of HFMD epidemics by year. The location of the primary clusters (dark blue) varied, and 3–4 clusters were identified each year. The epidemic typically started between weeks 17–24 (May-June) and lasted until week 29 (July) or 39 (September). In 2014 and 2016, when the HFMD cumulative incidence was higher than 10 per 100 persons, the most likely spatio-temporal cluster area was located in southeast Korea, and the epidemic occurred during the 17th week of 2014 and the 21st week of 2016. The coverage area was larger clustering in more than a 100 km radius and the risk was higher (RR 4.21 in 2014, RR 6.34 in 2016). Whereas in 2015, when the HFMD cumulative incidence was relatively low, the most likely spatio-temporal cluster (RR 2.32) was mainly located in south-central Korea, an area covering 65 counties, and clustered between the 17th to the 38th week of 2015. (Fig 2). In 2011, the most likely cluster covered 34 administrative districts, including parts of Seoul and northern Gyeonggi between weeks 22 and 30 (June–July) which clustered as high-risk areas (RR 5.68). Between 2012 and 2014, HFMD was prevalent from weeks 17–23 (May-June) to weeks 30–31 (July) in Busan, Ulsan, Gyeongbuk, and Gyeongnam. In 2015, when the HFMD incidence was relatively low, 65 administrative districts, including Daegu, Gwangju, Daejeon, parts of Jeonbuk and Jeonnam, and parts of Gyeongbuk and Gyeongnam, showed a low incidence between weeks 17 and 38 (RR 2.32). In 2016, when a large HFMD epidemic occurred, almost all district showed a sporadic HFMD epidemic. Busan, Daegu, Ulsan, and parts of Gyeongbuk and Gyeongnam clustered as high-risk areas (RR 6.34) between weeks 21 and 31, and 65 administrative districts surrounding Gangwon, including Seoul, Gyeonggi, and parts of Gyeongbuk (RR 4.03), as well as 65 districts around Chungnam and Jeolla (RR 3.63), showed an HFMD epidemic. In 2017, 32 districts around Gyeonggi and parts of Chungbuk had HFMD clusters (RR 4.23) between weeks 24 (June) and 36 (September), which was relatively seasonally later than previous years (Table 3, Fig 3).

**Table 3:** Results of the space-time cluster at the district level, 2011–2017.

Year	Cluster type	Cluster time (weeks)	Cluster centers/Radius	Counties (n)	RR	LLR	p-Value
**2011**	Most-likely	22–30 (9)	(37.51N, 127.57E) / 52.84 km	34	5.68	43566.1	<0.001
	Secondary 1	21–30 (10)	(37.45N, 126.66E) / 27.84 km	27	4.50	31043.0	<0.001
	Secondary 2	19–29 (11)	(35.82N, 129.23E) / 93.19 km	52	4.17	29283.0	<0.001
	Secondary 3	20–30 (11)	(34.81N, 126.04E) / 224.66 km	77	3.87	23268.4	<0.001
**2012**	Most-likely	23–31 (9)	(35.67N, 128.78E) / 70.93 km	52	4.66	18611.4	<0.001
	Secondary 1	23–31 (9)	(37.03N, 127.30E) / 53.68 km	33	4.00	13661.5	<0.001
	Secondary 2	23–31 (9)	(37.80N, 127.00E) / 35.10 km	31	2.92	6722.4	<0.001
	Secondary 3	21–31 (11)	(35.27N, 126.45E) / 152.38 km	62	2.85	5829.9	<0.001
**2013**	Most-likely	18–30 (13)	(35.82N, 129.23E) / 93.19 km	52	3.96	24029.3	<0.001
	Secondary 1	20–30 (11)	(37.40N, 126.91E) / 19.26 km	29	3.10	12331.0	<0.001
	Secondary 2	21-31(11)	(36.57N, 126.62E) / 71.93 km	23	3.04	7275.8	<0.001
**2014**	Most-likely	17–30 (14)	(35.01N, 128.29E) / 109.29 km	61	4.21	38310.4	<0.001
	Secondary 1	21–30 (10)	(37.70N, 128.83E) / 158.64 km	62	3.53	20369.7	<0.001
	Secondary 2	21–29 (9)	(37.71N, 126.40E) / 54.12 km	29	3.36	16670.4	<0.001
	Secondary 3	20–30 (11)	(36.57N, 126.62E) / 71.93 km	23	3.38	12566.4	<0.001
**2015**	Most-likely	17–38 (22)	(35.55N, 127.72E) / 97.78 km	65	2.32	7079.3	<0.001
	Secondary 1	18–36 (19)	(37.85N, 126.81E) / 40.56 km	32	2.03	4311.2	<0.001
	Secondary 2	18–38 (21)	(37.20N, 127.25E) / 35.82 km	25	2.07	4277.5	<0.001
**2016**	Most-likely	21–31 (11)	(35.07N, 129.06E) / 103.38 km	50	6.34	81906.6	<0.001
	Secondary 1	22–31 (10)	(37.70N, 128.83E) / 158.64 km	62	4.03	34188.8	<0.001
	Secondary 2	23–31 (9)	(35.67N, 126.64E) / 129.06 km	65	3.63	24160.4	<0.001
	Secondary 3	22–31 (10)	(37.28N, 126.69E) / 32.82 km	28	3.13	19603.7	<0.001
**2017**	Most-likely	24–36 (13)	(37.03N, 127.30E) / 52.85 km	32	4.23	23301.1	<0.001
	Secondary 1	24–39 (16)	(34.99N, 127.38E) / 143.03 km	76	3.38	17300.9	<0.001
	Secondary 2	24–37 (14)	(38.23N, 127.39E) / 90.70 km	40	3.12	13159.4	<0.001

RR; Relative Ratio, LLR; Likelihood Ratio, Cluster centers; Centroid (latitude, longitude)

**Fig 4:**
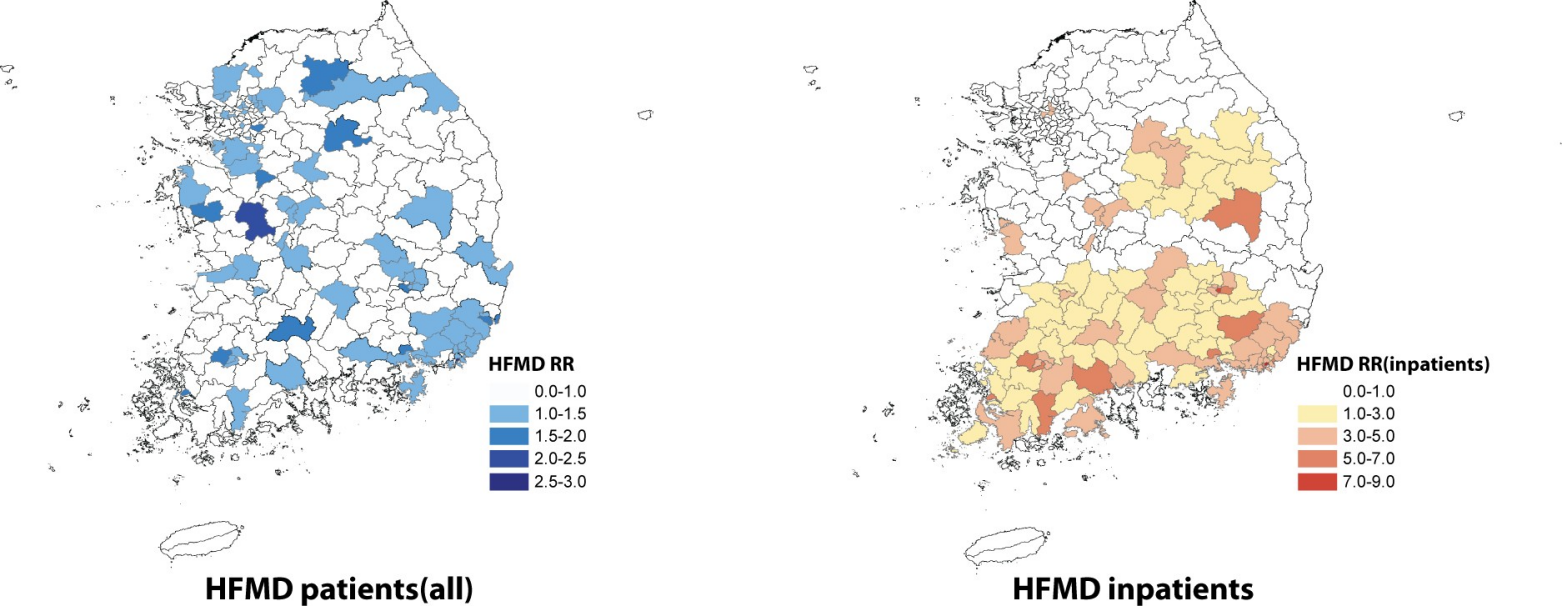
Relative risk maps for HFMD, 2011–2017.

An RR analysis of 2011–2017 showed the highest risk of incidence in Dong-gu, Busan (RR 2.94), Gongju, Chungnam (RR 2.36), Jung-gu, Busan (RR 1.96), Masan, Gyeongnam (RR 1.94), Nam-gu, Ulsan (RR 1.93), and Dong-gu Ulsan (RR 1.81), in descending order. Districts with a higher RR for HFMD-related hospitalization between 2011 and 2017 were Dong-gu, Busan (RR 7.97), Nam-gu, Daegu (RR 5.34), Suncheon, Jeonnam (RR 4.99), Gwangsan-gu, Gwangju (RR 4.95), Andong, Gyeongbuk (RR 4.95), and Milyang, Gyeongnam (RR 4.92) (Fig 4).

**Fig 5:**
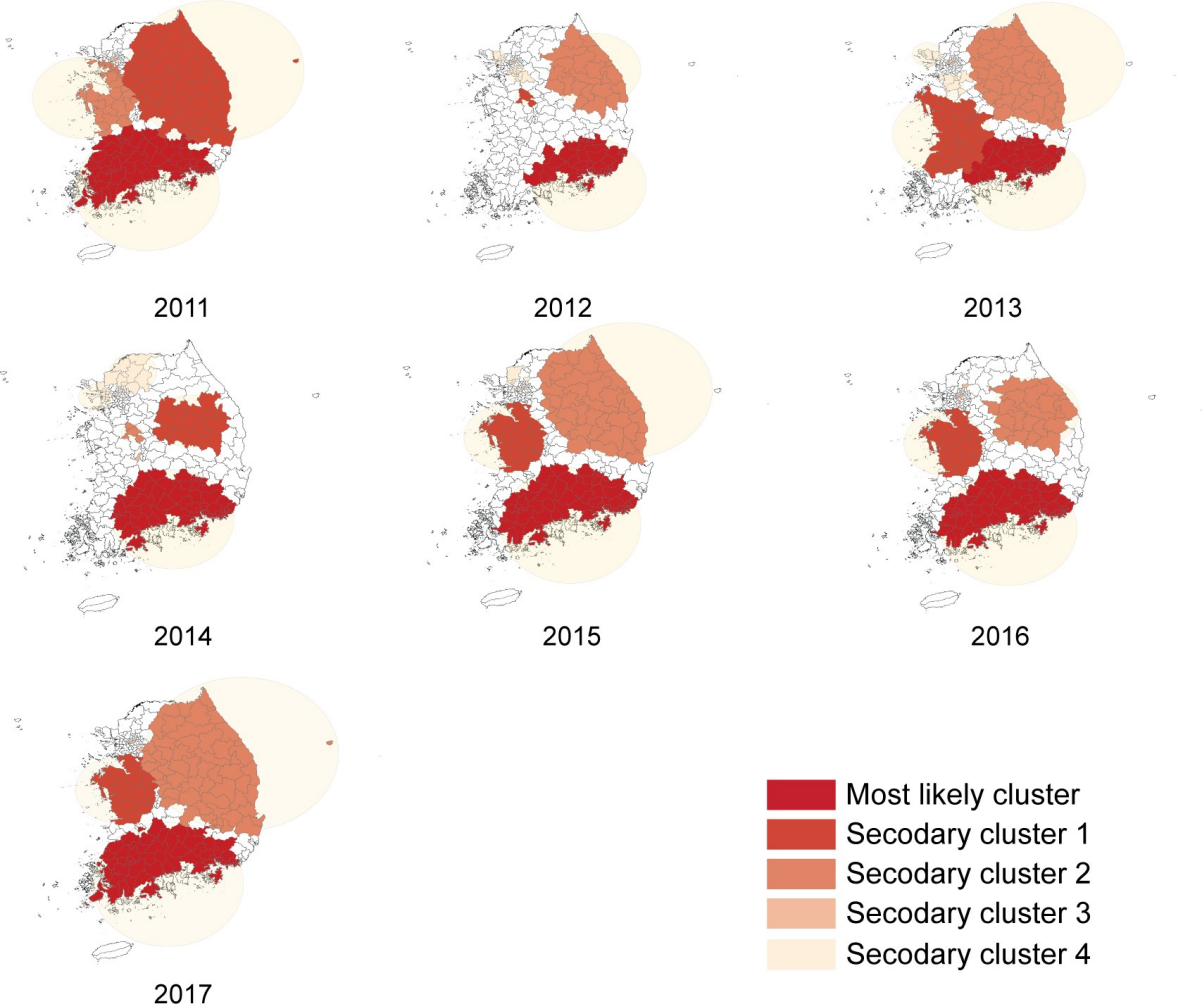
Spatiotemporal clusters of HFMD inpatients 2011–2017, setting 20% as the maximum cluster size.

In terms of hospitalization, the main clusters were located in Busan and Gyeongnam each year (dark red), and the clusters with the second-highest incidence varied year by year. The cluster time was 2–3 weeks earlier and lasted longer compared with the all-population HFMD incidence, including outpatients (Table 4, Fig 5).

**Table 4:** Results of the space-time cluster at the district level (inpatients), 2011–2017.

Year	Cluster type	Cluster time (weeks)	Cluster centers/Radius	Counties (n)	RR	LLR	p-Value
**2011**	Most-likely	18–30 (13)	(34.69N, 127.65E) / 142.51 km	75	8.50	1969.2	<0.001
	Secondary 1	22–31 (10)	(37.27N, 129.12E) / 169.59 km	63	5.01	638.4	<0.001
	Secondary 2	21–30 (10)	(36.70N, 126.28E) / 97.29 km	47	3.55	373.5	<0.001
	Secondary 3	21–30 (10)	(37.53N, 126.97E) / 6.29 km	6	6.71	233.8	<0.001
**2012**	Most-likely	20–35 (16)	(34.87N, 128.62E) / 113.27 km	55	7.69	1742.7	<0.001
	Secondary 1	22–34 (13)	(36.76N, 127.22E) / 18.55 km	3	7.84	357.5	<0.001
	Secondary 2	23–40 (18)	(37.37N, 128.73E) / 88.81 km	21	5.31	221.3	<0.001
	Secondary 3	23–41 (19)	(37.56N, 126.99E) / 3.49 km	2	6.86	83.1	<0.001
	Secondary 4	23–30 (8)	(37.27N, 127.05E) / 19.10 km	15	1.78	17.4	0.002
**2013**	Most-likely	17–33 (17)	(34.87N, 128.62E) / 115.89 km	56	6.89	2226.1	<0.001
	Secondary 1	21–37 (17)	(35.95N, 126.72E) / 96.49 km	46	2.92	383.7	<0.001
	Secondary 2	19–30 (12)	(37.50N, 129.05E) / 149.93 km	38	2.82	104.5	<0.001
	Secondary 3	21–31 (11)	(37.53N, 126.97E) / 6.23 km	5	3.46	70.5	<0.001
**2014**	Most-likely	16–40 (25)	(35.04N, 128.03E) / 109.21 km	66	7..77	3810.5	<0.001
	Secondary 1	21–30 (10)	(36.99N, 128.38E) / 57.76 km	13	5.48	172.5	<0.001
	Secondary 2	21–30 (10)	(36.76N, 127.22E) / 18.55 km	3	3.38	93.1	<0.001
	Secondary 3	20–28 (9)	(37.46N, 126.48E) / 27.95 km	11	1.65	22.6	0.0001
**2015**	Most-likely	14–39 (26)	(34.81N, 127.94E) / 127.20 km	70	6.28	1785.3	<0.001
	Secondary 1	18–22 (5)	(36.57N, 126.62E) / 71.93 km	23	3.57	116.0	<0.001
	Secondary 2	19–35 (17)	(37.50N, 129.05E) / 149.93 km	39	2.94	115.4	<0.001
**2016**	Most-likely	21–33 (13)	(34.81N, 127.94E) / 127.20 km	70	12.75	7602.0	<0.001
	Secondary 1	22–31 (10)	(36.57N, 126.62E) / 71.93 km	23	3.62	575.1	<0.001
	Secondary 2	21–30 (10)	(37.20N, 128.49E) / 78.98 km	20	4..75	282.4	<0.001
	Secondary 3	25–32 (8)	(37.53N, 126.97E) / 6.23 km	5	4.10	130.6	<0.001
**2017**	Most-likely	22–40 (19)	(34.69N, 127.65E) / 136.90 km	64	11.52	3604.8	<0.001
	Secondary 1	24–36 (13)	(36.57N, 126.62E) / 71.93 km	23	4.49	585.6	<0.001
	Secondary 2	26–31 (6)	(37.27N, 129.12E) / 169.59 km	63	2.98	153.1	<0.001

Cluster centers; Centroid (latitude, longitude)

## Discussion

This report aimed to explore the epidemiological characteristics as well as the temporal and geographic distribution of HFMD, which is a major cause of public health concern due to its high incidence in children and its high contagion rate. This report aimed to provide information for establishing national management and prevention strategies by predicting future incidence.

Male children and those younger than 4 years of age were found to be the most at risk for HFMD infection, which corresponded with other Asian studies. Although infection rates between males and females are comparable, males are more likely to develop symptoms, more involved in the propagation of outbreaks, and more likely to be brought in for medical care than females [8]. Over 90% of HFMD cases occur in children younger than 6 years of age, with the majority younger than 2 years of age. Therefore, measures must be taken to prevent HFMD in these population groups.

According to the data from the Western Pacific Region of the World Health Organization, in China, where complete monitoring of HFMD is conducted, HFMD epidemics peak around May–June, show a smaller peak in October, and occur every 2 years. Japan operates a sample monitoring system comprised of 3000 designated centers for HFMD. Their results show that HFMD incidences increase in May, peak around July–August, and occur every 3–4 years; similar to Korea [9].

HFMD in Korea shows a seasonal pattern, with incidences increasing from week 17 (May), peaking in week 26 (June), and continuing until week 39 (September). This pattern corresponds with results from northern China and Japan (two areas classified as HFMD epidemic areas), where the climate is relatively moderate for Western Pacific nations. Results from these studies showed that the highest incidence was between May and July [8–13]. The yearly incidence decreased in 2012 compared with 2011, and increased again in 2013–2014. Incidence was lowest in 2015, and a massive epidemic was observed in 2016 before decreasing again in 2017. This report’s results corresponded with HFMD sentinel surveillance system statistics results by the CDC (Infectious Disease Portal; http://www.cdc.go.kr/npt/)and had a high correlation coefficient of 0.97 [14]. In particular, in 2016, HFMD was highly prevalent, with 2 of 10 children younger than 2 years of age being affected. In terms of areas, Dong-gu in Busan were the highest in RR for HFMD, and the southern parts of the country showed a higher rate of hospitalization for HFMD. These results did not correspond to studies from other countries that showed higher incidences in large urban areas with higher population density [15–17]. This report recommends that to understand future incidences pattern of HFMD in Korea, such factors must be considered.

The major strength of the present study was the utilization of the national health insurance data (HIRA data), which covers the entire South Korean population without selection bias. By using HIRA data, it was possible to provide the occurrence of HFMD across age, sex, and region with the KCDC HFMD sentinel surveillance system.

However, the use of data from HIRA claims has limitations. Discrepancies in the data may occur between diagnoses and disease. A previous study that examined the accordance of diagnosis in HIRA data to the actual status of health conditions by comparing medical record reports showed that, on average, 70% of diagnoses corresponded to diagnoses in medical charts although the accordance rate was different depending on conditions, care settings and insurance providers [18]. To identify spatial clusters, we used health institutes locations rather than HFMD cases’ due to the unavailability of personal address in HIRA data. However, we believe most HFMD cases were not severe and visited local clinics near where they lived in. And the cluster size in this analysis was huge enough compared to the basic administrative regions we used in this data. So, the locations of the clusters in this analysis were considered quite robust despite this limitation.

## Conclusion

As the first study to explore epidemiological characteristics and to analyze the temporal and spatial distribution of HFMD in Korea, this report is meaningful. However, the findings in this report are limited by the nature of health insurance claim data, which are intended for medical expense claims and are less diagnostically accurate. Geographical distinctions were also made based on the location of the health care institution rather than the patients’ areas of residence. This distinction could cause some regional bias, even though patients do not typically seek medical care for HFMD far from home.

## Supporting information

S1 Table Spatio-temporal clusters of hand-food and mouth disease and relative risks by district and year (2011–2017). (XLSX)
